# Phosphatidylcholine-Polysorbate 20-Based Mixed Micelles: A New Option to Prevent Protein Aggregation?

**DOI:** 10.3390/pharmaceutics18030321

**Published:** 2026-03-02

**Authors:** Johanna Weber, Tim Diederichs, Lukas Bollenbach, Patrick Garidel, Karsten Mäder

**Affiliations:** 1 Institute of Pharmacy, Faculty of Biosciences, Martin Luther University Halle-Wittenberg, Wolfgang-Langenbeck-Strasse 4, 06120 Halle (Saale), Germany; johanna.weber@pharmazie.uni-halle.de (J.W.); lukas.bollenbach@boehringer-ingelheim.com (L.B.); 2 TIP, Innovation Unit, Boehringer Ingelheim Pharma GmbH & Co. KG, Birkendorfer Straße 65, 88400 Biberach an der Riss, Germany; tim.diederichs@boehringer-ingelheim.com (T.D.); patrick.garidel@boehringer-ingelheim.com (P.G.); 3 Physical Chemistry, Institute of Chemistry, Martin Luther University Halle-Wittenberg, Von-Danckelmann-Platz 4, 06120 Halle (Saale), Germany

**Keywords:** biotherapeutic formulations, biopharmaceuticals, mixed micelles, alternative surfactants, polysorbates, phospholipids, protein stabilisation, shear stress

## Abstract

**Background/Objectives:** Surfactants are commonly used to protect proteins from denaturation and particle formation, thereby ensuring the long-term stability of biopharmaceuticals. Polysorbates (PS) 20 and 80 are the most widely used surfactants in the pharmaceutical industry. However, alternative excipients such as poloxamers are currently under investigation. In this study, mixed micelles (MMs) composed of phospholipids (PL) and polysorbate 20 (PS20) were explored as a novel stabilisation strategy, aiming to reduce the PS content in protein formulations by partial substitution with PL. Despite their favourable properties, including thermodynamic stability and small particle size, MMs have seen limited application, and no reports exist on their use for stabilising antibody solutions. **Results:** In a first step, PS20/PL ratios were identified, which are advantageous to form stable MM solutions, followed by an optimization of the formulation process by introducing a second heating step using the direct dispersion method. Successful MM formation was confirmed via transmission and dynamic light scattering analyses at total surfactant concentrations of up to 20 mg·mL^−1^ and 50 mg·mL^−1^, with PL contents of 50% and up to 40%, respectively. These surfactant concentrations of up to 20 mg·mL^−1^ and 50 mg·mL^−1^ are substantially higher than the surfactant concentrations that are typically used in final biopharmaceutical formulations (0.01–2 mg·mL^−1^). Consequently, the mixed micellar system enables operation even at concentrations substantially above practical formulation limits. In the ensuing study, the stabilizing potential of the PL/PS20 micellar system was appraised through agitation studies. **Methods:** In these studies, bovine serum albumin was employed as a model protein, while a monoclonal antibody was used as a candidate therapeutic molecule. Stability was assessed through visual inspection, turbidity measurements, particle analysis, and size-exclusion chromatography. **Conclusions:** A protective effect comparable to that of PS20 alone was observed for both model proteins, demonstrating for the first time that MMs can effectively stabilise biologics.

## 1. Introduction

Protein damage, causing the formation of high molecular weight (HMW) species and protein particles, is a severe issue during the manufacturing of biopharmaceuticals [1]. Surfactants are frequently used to protect protein active pharmaceutical ingredients against particle formation by saturating hydrophobic interfaces [2]. Non-ionic surfactants such as polysorbate 20 (PS20) and polysorbate 80 (PS80) are most commonly used to protect monoclonal antibodies (mAbs). It is known that polysorbates (PSs) are complex mixtures of several species with different properties [3,4,5]. Recently, many research efforts have been made to understand the details and mechanisms of their oxidative and hydrolytic degradation pathway [3,6,7,8,9]. In particular, the enzymatic degradation caused by host cell proteins bears the risk of particle formation due to ester bond cleavage and release of free fatty acids [10,11,12,13,14,15,16,17,18,19,20,21,22,23,24,25,26,27].

Therefore, the demand for PS substitutes is still strong. Proposed alternatives are poloxamers [28,29], cyclodextrins [29,30,31,32], Brij’s [33,34,35], or entirely novel excipients such as FM1000 [36,37,38,39]. Poloxamer 188 (P188), which is a non-ionic triblock copolymer consisting of 25 to 30 poly(propylene oxide) groups flanked by two blocks of 75 to 85 poly(ethylene oxide) moieties [28]. It is, up to now, the only alternative surfactant used in FDA-approved protein formulations [9,38]. Nevertheless, P188 possesses oxidation potential as well. Additionally, it bears the risk of protein-polydimethylsiloxane particle formation in formulations in contact with silicon surfaces, which might be critical for highly concentrated protein formulations in prefilled syringes [40,41,42,43]. As an alternative to the complete replacement of PS, a viable strategy is to reduce the PS fraction and partially substitute it with an endogenous surfactant, specifically a phospholipid (PL), by formulating mixed micelles.

PLs are widely used as emulsifiers in food, cosmetics, and pharmaceuticals [44,45]. Within parenteral drug delivery, they are used as excipients for liposomal drug delivery and stabilising parenteral lipid emulsions. Moreover, PLs are known for their excellent biocompatibility and safety. Nevertheless, they have not been used as stabilisers within protein formulations so far. Depending on their molecular structure, PLs form vesicles (liposomes) rather than micelles in water due to their molecular geometry [46]. Phospholipids dispersed as vesicles will not be able to cover new surfaces rapidly because of the low monomer content in the aqueous phase. However, mixtures of PLs with surfactants can form mixed micelles (MM) depending on surfactant composition and manufacturing method. MM are, compared to liposomes, smaller and more flexible. Hans Steffen has done pioneering work on using MMs in pharmaceutical solutions as a carrier for small-molecule drugs [47]. Commercial MM products were developed, composed of bile acid and PLs, which were used for oral and parenteral administrations for poorly soluble small-molecule drugs such as Diazepam (Valium MM), Lenvatinib, Carprofen (veterinarian), Vitamin A, D, E, or Vitamin K (Konakion^®^MM) [48,49,50]. Due to their small size, MMs form transparent systems [51,52].

Until now, no applications using MMs for stabilising protein formulations are known. Therefore, the current study aimed to explore the potential of MMs for protein stabilisation. This work addresses the following main questions: (i) Are MMs able to prevent protein aggregation induced by shaking stress? (ii) Can PS20 MMs prevent protein aggregation to a similar level as PS20 only? (iii) Is it possible to reduce the total amount of PS20 by using MMs?

MMs possess properties differing from those of the individual surfactants [53,54,55]. Specific characteristics of MMs, like foaming, rheological properties, shape, size, or the critical micelle concentration (CMC), can be adjusted in contrast to the individual surfactants [56,57,58,59]. In particular, the CMC may be used as a surrogate parameter to assess the coverage of hydrophobic interfaces. Surfactants showing lower CMC values require smaller amounts for saturating hydrophobic interfaces [60]. Therefore, they potentially protect proteins against denaturation and HMW species formation at lower concentrations [1].

To investigate the potential of MM for protein stabilisation, at first, different conditions and PS20-PL ratios for generating stable MM solutions were tested. Based thereon, this study focuses on MMs with a comparably high PL/low PS content and a robust MM formation process. To ensure complete solubilisation of the PL within the MMs during manufacturing, heating above the phase transition temperature (T_m_) of the lipid is essential [52,61]. The T_m_ of a phospholipid depends on its headgroup, carbon chain length, and the degree of saturation. To avoid oxidation problems linked with unsaturated fatty acids, we used the hydrogenated phospholipid SPC3 (Lipoid, Ludwigshafen, Germany) for our studies. It is suitable for parenteral use and contains approximately 13% palmitic acid (C16:0), 86% stearic acid (C18:0), as well as <1% oleic acid (C18:1) [62]. SPC3 has a phase transition temperature of approximately 54 °C, determined via differential scanning calorimetry (DSC) measurements.

Different methods for preparing mixed-micellar systems are known, such as the direct dispersion method, which avoids using organic solvents [50,61,63,64]. By mixing the water-soluble detergent with the water-insoluble lipid at elevated temperature (above the thermotropic transition temperature of the lipid), followed by an equilibration step at 37 °C or 25 °C under stirring, MMs with different phosphatidylcholine (PC) weight fractions (wf) of 0 to 0.7 were formed by the group of Rupp [50,61]. A disadvantage was the long processing time of several hours.

It is therefore the aim of this study to optimise the MM formation process and to investigate the protein stabilisation properties of PS/PL MM compared to PS20 solely.

## 2. Materials and Methods

### 2.1. Materials

Milli-Q^®^ ultrapure water, purified by an IQ 7005 Purification System, Merck KGaA (Darmstadt, Germany), was used. A monoclonal antibody (mAb1) was used as a model protein. mAb1 was produced by Boehringer Ingelheim (Biberach an der Riss, Germany) according to the described processes [65]. Polysorbate 20 high purity (HP) and polysorbate 80 HP were purchased from Croda International (Snaith, UK). The phospholipid (hydrogenated soybean phosphatidylcholine SPC3, maximal iodine value of 3) was purchased from Lipoid (Ludwigshafen, Germany). Acetic acid (99–100%) was purchased from Honeywell International Inc. (Charlotte, NC, USA), sodium acetate trihydrate and ortho-phosphoric acid were purchased from Merck KGaA (Darmstadt, Germany), isopropanol, L-arginine, and ammonium sulphate for size-exclusion chromatography (SEC) analysis were purchased from Sigma-Aldrich (St. Louis, MO, USA). Bovine Serum Albumin was purchased from Sigma Aldrich, St. Louis, MO, USA.

### 2.2. Methods

#### 2.2.1. MM Preparation: Optimised Direct Dispersion Method (5-2-2 Method)

Different weight ratios (weight fraction of 0.3 to 0.6) of PS20 and the phospholipid SPC3 were mixed to final concentrations of 5 mg·mL^−1^, 10 mg·mL^−1^, 20 mg·mL^−1^, and 50 mg·mL^−1^ in water or the corresponding buffer (acetate, citrate, or histidine buffer, pH 5.5). First, the solutions were stirred with a thermostatic magnetic stirrer for 5 h at 55 °C, followed by a cooling step to 25 °C and stirring for another 2 h. Subsequently, a second heating step for 2 h at 55 °C under constant stirring was performed. This heating for initially 5 h under stirring at 55 °C, followed by a cooling and stirring step to 25 °C, followed by the second heating and stirring step at 55 °C for 2 h, is abbreviated as the 5-2-2 method. The evaluation revealed that in the context of this production method, clear (>99% transmission) and monodisperse MM solutions were obtained after the second heating step. A sample was taken every hour to evaluate the formation of MMs, and transmission and particle sizes were measured. The temperature of 55 °C was chosen to enable the hydration of the phospholipid above its thermotropic transition temperature (which was determined to be approx. 54 °C for SPC3).

#### 2.2.2. Shaking Studies of Protein Formulations

Two model proteins were tested within the shaking studies, namely, bovine serum albumin (BSA) and mAb1. Protein concentrations of 10 mg·mL^−1^ (mAb1) and 5 mg·mL^−1^ (BSA) were formulated with either MMs (fixed wf_SPC3_ of 0.3) or the corresponding pure polysorbate (PS) at a final surfactant (MM or PS) concentration of 0.2 mg·mL^−1^ in ultra-pure water or acetate buffer (25 mM and pH 5.5). The MMs were prepared using the methods described above. Within albumin shaking studies, 2.5 mL of the respective formulation was filled into 2R vials. For mAb1 agitation studies, 2R vials were filled with 1 mL of the different formulations and were 3D-shaken at 64 rpm in a Turbula^®^ T2F shaker from Fa. Willy A. Bachhofen AG (Basel, Switzerland) for up to 64 h at room temperature. Samples were investigated initially after 24 h (BSA), and after 40 h and 64 h (mAb1). BSA-containing samples were only used as initial trials in MM_PS20_ experiments. These were analysed via visual inspection, transmission, and DLS after shaking. All samples containing mAb1 were analysed via visual inspection, turbidity, size-exclusion chromatography, and particle analysis after shaking.

#### 2.2.3. Dynamic Light Scattering (DLS)

To monitor particle size distribution during the MM manufacturing process, DLS measurements were conducted (Zetasizer NanoZS from Malvern; He-Ne laser at 633 nm, Worcestershire, UK) using disposable UV cuvettes from Brand GmbH & Co. KG (Wertheim, Germany). The temperature was set to 25 °C with a 60 s equilibration time. The dispersant was set to water with a viscosity of 0.8872 mPa·s and a refractive index of 1.330. The measurement was conducted in 173° backscattering mode with 11 runs and a 10 s run duration. Measurements were performed in triplicate (*n* = 3). Size and polydispersity index (PDI) were determined. Intensity-weighted sizes are shown, whereas the size with the highest intensity is displayed within the figures. To avoid artefacts, samples were not filtered before measurements. Before analysis, samples were covered with parafilm to prevent dust particles from entering the solutions and were immediately measured.

For the dilution experiments on the MMs, a Prometheus Panta (NanoTemper Technologies, Inc., South San Francisco, CA, USA) was used to determine the particle size in solutions. The samples were filled into high-sensitivity capillaries (Prod. No. PR-C006; NanoTemper Technologies, Inc., South San Francisco, CA, USA) as triplicates and measured using the Panta Control software (Version 1.7.4, NanoTemper Technologies, Inc., South San Francisco, CA, USA) in the size determination mode. All measurements were conducted in the high sensitivity mode (10 measurements, 5 s) at 100% laser power. The temperature was set to 25 °C. The data was analysed using Panta Analysis software (Version 1.7.4, NanoTemper Technologies, Inc., South San Francisco, CA, USA).

#### 2.2.4. Transmission Measurement

A Lambda 35 UV/Vis spectrophotometer from Perkin Elmer (Waltham, MA, USA) was used to monitor the transmission during MM preparation. The absorbance at 660 nm was recorded, followed by conversion to transmission. Therefore, a 200 µL sample volume was measured in 10 mm single-use micro cuvettes from Brand GmbH & Co. KG (Wertheim, Germany) in duplicates (*n* = 2).

#### 2.2.5. Visual Inspection (VI)

VI was conducted with each vial at each sampling time point before and after shaking according to European Pharmacopoeia (Ph. Eur. 10.3), swirling every vial for 5 s before inspecting it in front of an inspection table’s black and white background. Every vial was cleaned with a dust-free tissue before inspection. Photos were taken with a Canon EOS 850D after a 30 min rest phase. Sections of every vial were extracted from the original images and placed in logical order for better visualisation of the corresponding figures.

#### 2.2.6. Turbidity

Turbidity measurements were conducted using a custom-made turbidity photometer (Boehringer Ingelheim, Biberach, Germany, and Microparts, Dortmund, Germany) as a complementary technique to VI. The results were given in formazin nephelometric units (FNU) in a calibration range between 0 and 100 FNU. 120 µL samples were filled into 15 mm single-use glass cuvettes. The measurements were performed based on scattered light of 633 nm at an angle of 90 °C in duplicates (*n* = 2).

#### 2.2.7. Ultra-Performance Size-Exclusion Chromatography (UP-SEC)

UP-SEC analysis was performed to quantify the monomer content, low molecular weight (LMW) species, and HMW species of the antibodies before and after shaking (agitation). SEC analysis was performed with an Acquity UPLC H-Class System from Waters (Milford, MA, USA) coupled to an ultraviolet diode array detector that detected absorbance at 280 nm. A volume of 5 µL, containing a theoretical sample load of 50 µg protein, was injected into an Acquity UPLC column BEH200SEC (300 × 4.6 mm, 20 nm) from Waters (Milford, MA, USA) with a KrudKatcher Ultra HPLC In-Line Filter (0.5 µm × 0.004 µm ID) from Phenomenex (Aschaffenburg, Germany) at 5 °C with a flow rate of 0.2 mL·min^−1^. The mobile phase contained 200 mM (3.48%) l-arginine, 120 mM (1.59%) ammonium sulphate, and 10% isopropanol in water. The pH was adjusted to 7.3 with ortho-phosphoric acid. Before measurement, all samples were centrifuged with a Fisherbrand^TM^ Mini-Centrifuge (Waltham, MA, USA) for 300 s. Data were evaluated with the Empower™ software (Empower 3 Feature Release 5 (FR5)). The amount of HMW is given as the percentage of the initial measured sample of the chromatogram. System suitability was validated via a functional protein standard.

#### 2.2.8. Backgrounded Membrane Imaging (BMI)

BMI measurements were conducted for analysing sub-visible particles > 2 µm using a HORIZON device (Halo Labs, Burlingame, CA, USA) under a Laminar Air Flow [66]. Following the background acquisition, a sample volume of 30 µL was loaded. To reduce the risk of an overloaded membrane, samples were diluted 1:100 in MilliQ^®^ water under laminar air-flow conditions. Two washing steps were performed using particle-free deionised water. The liquid was removed by vacuum filtration until the membrane surface was completely dry. The unloaded wells and the washing solution were subjected to further analysis to ascertain the absence of contamination [66]. The data were analysed using the Horizon VUE software v2.0 (Halo Labs, Burlingame, CA, USA).

#### 2.2.9. Differential Scanning Calorimetry (DSC)

To obtain the phase transition temperature of phospholipid (SPC3) in an aqueous environment, DSC measurements were conducted via a Mettler Toledo DSC 821e (Columbus, OH, USA) using approx. 10 mg SPC3. Before measurement, the PL was incubated in MilliQ^®^ at ambient temperature for 30 min for swelling and subsequently homogenised for 2 min with an IKA^®^ DI18 Basic yellow line homogeniser (Staufen, Germany) at 14,000 rpm. The ratio of MilliQ^®^ to SPC3 was set to 3:1. A closed aluminium pan (*n* = 3) was heated from 25 to 75 °C using a heating rate of 0.5 K·min^−1^ to characterise the thermal behaviour of the SPC. The maximum of the second heating curve was identified as the phase transition temperature.

#### 2.2.10. Fluorescence Experiments with Nile Red

A Quanta Master 4 CW (HORIBA Europe GmbH, Oberursel, Germany) controlled by PTI Felix 32 was used for the fluorescence measurements. 2.5 mL samples were filled into disposable fluorescence cuvettes (Carl Roth GmbH + Co. KG; Karlsruhe, Germany) and tempered to 25 °C using a T 400 temperature controller (Quantum Northwest, Inc., Liberty Lake, WA, USA) with a tempered cuvette holder. As described by Bollenbach et al., a 1 mM stock solution of Nile Red in methanol was prepared [67]. 25 µL of the stock solution was dissolved in 9.98 mL of the appropriate solvent. A volume of 0.5 mL of these solutions was mixed with the surfactant solutions to obtain samples with specific Epplersurfactant concentrations and 0.5 µM Nile Red. Fluorescence spectra were recorded between 580 nm and 780 nm with an excitation wavelength of 550 nm. The ratio of the fluorescence intensity at 618 nm and 645 nm emission wavelength was calculated and used for graphical representation.

#### 2.2.11. Data Evaluation

Statistical analyses were performed using Origin (OriginPro 2024b, version 10.1.5.132, OriginLab, Northampton, MA, USA) and Microsoft Excel (Version 2508, Microsoft Corp., Redmond, WA, USA). Data are reported as mean ± standard deviation (SD) unless otherwise stated. The number of biological and technical replicates (*n*) is indicated in each figure legend.

## 3. Results

### 3.1. Evaluation of an Optimised Dispersion Mixed Micelle Production Method—The 5-2-2 Method

The direct dispersion process for producing MMs was described nearly half a century ago. It was adopted by Rupp and colleagues, who generated MMs containing different surfactants (including PS20 and PS80) and PLs [50,61,68]. These surfactants were mixed with PLs in aqueous solution and stirred for 5 h at temperatures above the lipids’ transition temperature (55 °C), followed by equilibration at 25 °C or 37 °C overnight [50,61]. In the present study, this procedure was optimised. The final protocol of the optimisation process is a result of numerous and tedious experiments with different time and temperature profiles. The best results were achieved by shortening the equilibration step at 25 °C or 37 °C to 2 h, and by adding a second heating step at 55 °C for 2 h. This new method, the 5-2-2 method, denotes a stirring protocol consisting of 5 h of stirring at 55 °C, 2 h of stirring at 25 °C, and a final stirring of 2 h at 55 °C. It is therefore abbreviated as the 5-2-2 method. To determine whether this procedure resulted in clear or turbid samples and thereby indicated successful or incomplete micellization, transmission measurements were performed at 660 nm to provide a rapid, quantitative assessment of turbidity. Supplement Figure S1 exemplarily illustrates the beneficial effect of the second heating step for the MM production in acetate, citrate, and histidine buffer. The transmission values for solutions of 50 mg·mL^−1^ total surfactant concentration at a wf_SPC3_ of 0.4 are shown. Depending on the used buffer system, transmission values ranging between 65% and 75% were measured after the initial heating to 55 °C, and transmissions between 65% and 82% after 12 h at 37 °C (Supplement Figure S1). However, clear solutions with transmission values ≥ 98% after 1 h of additional heating to 55 °C were consistently observed under the tested conditions, highlighting the role of the additional heating step with temperatures above the lipid transition temperature (Supplement Figure S1). The adapted 5-2-2 method was evaluated with an equilibrium phase at 25 °C and 37 °C, while no differences regarding the formation of clear MM solutions were observed. Therefore, MMs in all further experiments were prepared with an equilibration phase at 25 °C.

**Figure 1:**
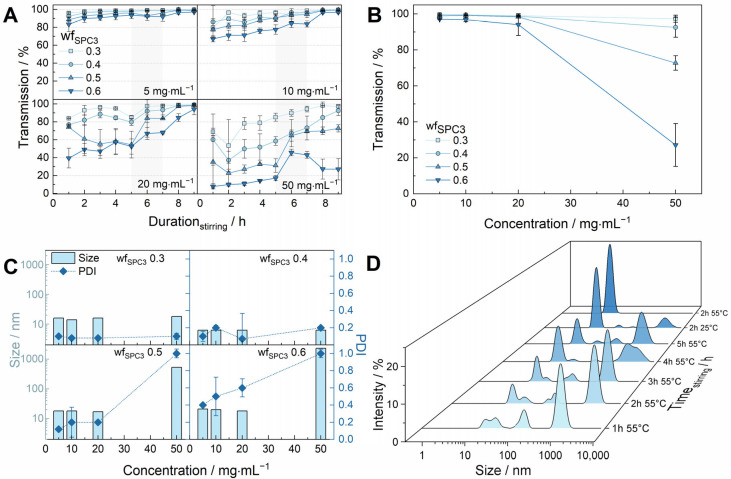
Evaluation of the optimised MM formation method (5-2-2 method) for different total MM concentrations and phospholipid weight fractions in an acetate-buffered solution, pH 5.5. MMs composed of PS20 and phospholipid (SPC3) were prepared using the described optimised 5-2-2-method for total mixed micelle concentrations of 5 mg·mL^−1^, 10 mg·mL^−1^, 20 mg·mL^−1^, and 50 mg·mL^−1^ and phospholipid weight fractions (wf_SPC3_) ranging from 0.3 to 0.6. Three technical replicates (*n* = 3) were produced and measured. (**A**) Transmission values for the different concentrations and wf_SPC3_ were determined for different time intervals within the method (**B**) The transmission values of MMs after 9 h (completion of the 5-2-2 method) are given for different total MM concentrations and wf_SPC3_ (**C**) The hydrodynamic diameter and the polydispersity index (PDI) for different wf_SPC3_ were plotted per total MM concentration (**D**) The size distribution (intensity weighted) for 20 mg·mL^−1^ and wf_SPC3_ of 0.4 for different time intervals within the 5-2-2 method is plotted.

To characterise the 5-2-2 method, different wf_SPC3_ ranging from 0.3 to 0.6, as well as different total MM concentrations (5 mg·mL^−1^, 10 mg·mL^−1^, 20 mg·mL^−1^, and 50 mg·mL^−1^) were investigated using transmission and DLS measurements for the production of MMs in acetate buffer, pH 5.5 (Figure 1). Simply mixing the PL and PS20 (first 5 h at 55 °C) did not yield fully transparent solutions under the tested conditions (Figure 1A). For total MM concentrations of 20 mg·mL^−1^, the transmission data varied between 40% and 84% for the different PL weight fractions after 1 h at 55 °C (Figure 1A). For 50 mg·mL^−1^ MMs, initial transmission values were even lower, ranging between 8% transmission for wf_SPC3_ of 0.6 and 69% for wf_SPC3_ of 0.3 (Figure 1A). The solutions became more transparent with increasing time, which is especially obvious for higher MM concentrations of 50 mg·mL^−1^ (Figure 1A). Transmissions of ≥ 99% were observed under most tested conditions after 9 h, except for the higher total MM concentrations in combination with high PL weight fractions, such as 20 mg·mL^−1^ and wf_SPC3_ of 0.6, or 50 mg·mL^−1^ with wf_SPC3_ of 0.5 or 0.6 (Figure 1A,B). By increasing the total MM concentration and the PL weight fractions, the impact of the second heating step can be observed, as the solutions became transparent after heating up to 55 °C for the second time (Figure 1A). The resulting transmission values are shown for different total concentrations and different weight fractions of SPC3 after using the 5-2-2 method (Figure 1B). Additionally, the hydrodynamic diameter of the MMs, as well as the corresponding PDI, were analysed by DLS after the formation of MMs with the 5-2-2 method. Similar trends were observed to those detected by transmission analysis (Figure 1C). For total MM concentrations of up to 50 mg·mL^−1^ and wf_SPC3_ of 0.3 and 0.4, hydrodynamic diameters of approximately 15 nm and PDIs ≤ 0.2 were determined for all conditions (Figure 1C). Similar results were obtained with MMs formed with wf_SPC3_ of 0.5 and total MM concentrations up to 20 mg·mL^−1,^ which also revealed diameters of approximately 15 nm with PDI values ≤ 0.2 (Figure 1C). However, for total micelle concentrations of 50 mg·mL^−1^ and weight fractions of SPC3 of 0.5 and 0.6, larger particles (>500 nm) and very high PDI values were observed. The particle sizes of total concentrations of 5, 10, and 20 mg·mL^−1^ and wf_SPC3_ 0.6 were still below 20 nm, however, showing high PDI values of 0.4–0.6 (Figure 1C). Size distributions throughout different time intervals of the 5-2-2 method for total micelle concentrations of 20 mg·mL^−1^ and wf_SPC3_ of 0.4 are shown in Figure 1D. A multimodal particle size distribution was observed up to the second heating step, which changes into a monomodal size distribution with a hydrodynamic diameter of approx. 15 nm after completion of the second heating step. This reveals the effective solubilisation of the PL within the MM (Figure 1D).

### 3.2. Shaking Studies of Mixed Micelles with BSA

**Figure 2:**
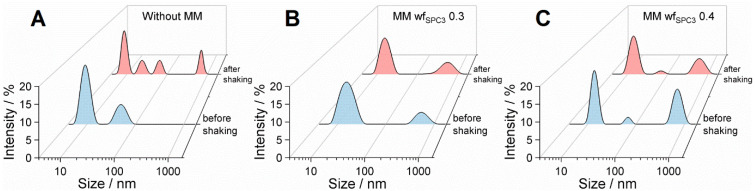
Particle size distributions of bovine serum albumin (BSA) solutions used in a shaking study. BSA solutions were spiked with MMs composed of PS20 and phospholipid (SPC3) in weight fractions of lipid (wf_SPC3_) of 0.3 (**B**) and 0.4 (**C**), as well as without surfactant (**A**), in a 25 mM acetate buffer pH 5.5, and were shaken in a Turbula^®^ shaker at 64 rpm for 24 h at RT. The surfactants were used at final concentrations of 20 mg·mL^−1^. Particle size distributions were analysed before shaking, as well as after shaking without surfactants and with MMs. BSA concentrations of 5 mg·mL^−1^ were used.

The prevention of shaking stress-induced protein aggregation by MMs composed of PS20 and PL SPC3 was first evaluated with the model protein BSA. Therefore, 5 mg·mL^−1^ BSA formulations were mixed with MMs (final concentration of 20 mg·mL^−1^ and wf_SPC_ of 0.3 and 0.4) in 25 mM acetate buffer, pH 5.5, and were shaken in a Turbula^®^ shaker with 64 rpm for 24 h at RT. MMs were produced using the 5-2-2 method as described above. BSA was shaken without surfactant as a positive control for the shaking stress. All samples were analysed using DLS measurements to detect emerging particles after shaking and evaluate if the MM system can prevent or minimize protein-aggregation. Intensity-weighted particle size distributions are illustrated in Figure 2 for visualisation of larger particle populations. Before shaking, a main peak at approximately 10 ± 2 nm (peak maximum ± standard deviation) and a second small peak at approximately 52 ± 12 nm were observed via DLS for the albumin solution without surfactants (Figure 2A). After shaking in the absence of surfactant, two additional peaks were observed, with an emerging peak at approximately 698 ± 63 nm, as well as another peak at 24 ± 4 nm in addition to the main peak at 9 ± 1 nm (Figure 2A). These emerging peaks most likely indicate the formation of larger protein particles. In samples containing MM wf_SPC3_ 0.3, the particle size distribution after shaking was similar to the initially measured size distribution with a main peak at 16 ± 5 nm. (Figure 2A). Formulations containing MM with a wf_SPC3_ of 0.4 showed comparable trends, as similar particle size distributions were observed before and after shaking. A main peak was found at 14 ± 2 nm, with two additional peaks that did not change much after shaking. Both formulations containing MMs showed higher particle size values than the main peak without surfactant. The data suggest that MMs, regardless of the tested PL concentrations, provide a preventive effect under the tested conditions. Based on this, further shaking studies were performed with mAb1.

### 3.3. Detection of the Mixed-Micellar System and Mixed-Micellar Integrity During Dilution

**Figure 3:**
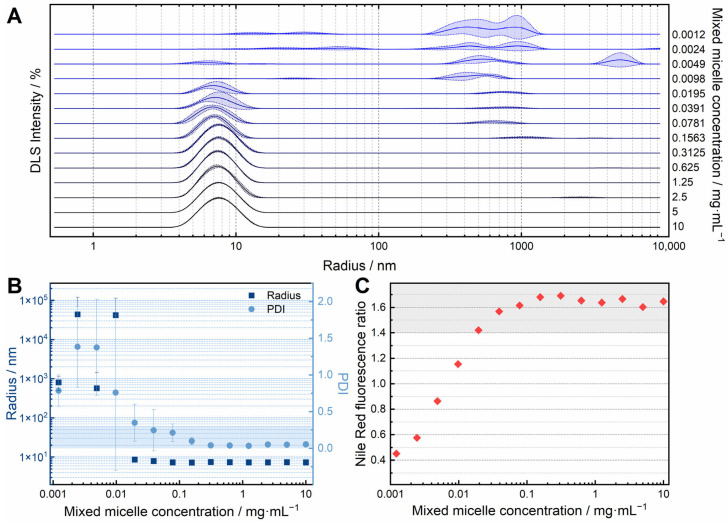
Results from dynamic light scattering (DLS) and Nile Red fluorescence experiments demonstrate the existence of PS20 mixed micelles. MM_PS20_ at a concentration of 10 mg·mL^−1^ and with a wf_SPC3_ of 0.3 were prepared in pure water using the 5-2-2 method. A 1:1 dilution series with water was prepared. (**A**) Intensity-weighted size distributions determined using DLS. Hydrodynamic radii of 7–8 nm indicate the presence of mixed micelles. (**B**) Hydrodynamic radii (z-average) and PDI values for the diluted mixed micelle samples. A PDI of ≤ 0.3 (shaded blue) indicates a predominantly monodisperse size distribution. (**C**) Results of the Nile Red solubilisation experiments. The procedure and quotient formation were carried out in the same way as described in the literature [67]. In the case of the mixed micelles, the ratio of the intensity at 618 nm and 645 nm was used. A ratio of ≥ 1.4 (shaded gray) indicates the formation of a hydrophobic compartment through mixed micelles in solution.

To confirm that the method described predominantly produces MMs and not vesicular structures, such as liposomes, dynamic light scattering measurements were performed. A dilution series of the MMs was examined by DLS (Figure 3A,B) to determine the presence of MMs in diluted samples. In the intensity-weighted size distributions (Figure 3A) at concentrations between 10 mg·mL^−1^ and 0.0195 mg·mL^−1^, maxima in the distributions can be observed at radii between 7 nm and 8 nm. Only when further diluted to concentrations below 0.0195 mg·mL^−1^, those intensity peaks vanish in favour of non-specific signals between 100 nm and 1000 nm radii sizes. The observations from the size distribution curves are also reflected in the z-average and PDI values calculated by the software (Figure 3B). For concentrations between 10 mg·mL^−1^ and 0.0195 mg·mL^−1^, radii of less than 10 nm (7–9 nm) were calculated, whereby the PDI is always less than 0.4, and between 0.0391 mg·mL^−1^ and 10 mg·mL^−1^, even less than 0.3. At concentrations lower than 0.0195 mg·mL^−1^, the radius is quantified as >100 nm and the PDI as >0.5, indicating the formation of vesicles or vesicle aggregates.

In addition to monitoring the particle radii, the existence of a hydrophobic compartment characteristic of micelles within the solution was also investigated to assess the presence of MMs. A fluorescence method was used with the dye Nile Red, which was successfully used to detect poloxamer 188 micelles [67]. By forming a ratio of two intensity maxima of Nile Red at 545 nm and 518 nm, the presence of a hydrophobic compartment can be demonstrated. With ratio values of >1.4, micelles are indicated to be present in the solution under these conditions. Figure 3C shows that the ratio of the two intensities decreases steeply below a concentration of 0.0195 mg·mL^−1^. At concentrations of 0.0195–10 mg·mL^−1^, however, all values are in the range characteristic of micelles (>1.4).

### 3.4. Shaking Studies with a Monoclonal Antibody

To further evaluate MMs’ protective protein stabilisation properties, a shaking study was performed with mAb1 in water and acetate buffer pH 5.5. The mAb1 concentration was set to 10 mg·mL^−1^, and MMs were similarly prepared as in the BSA studies but with a final total MM concentration of 0.2 mg·mL^−1^ and a lipid weight fraction of wf_SPC3_ 0.3. Previously shaking conditions with a 2R vial filled with 1 mL formulation were considered suitable for stress testing [69]. Therefore, the fill volume in the 2R vials was set to 1 mL to provide a large headspace and proper interaction with interfaces [70]. In this study, formulations were shaken at RT for 40 h and 64 h at 64 rpm using a Turbula^®^ shaker. Formulations in vials were visually inspected (VI), and transmission and turbidity measurements were performed to complement the VI results. Additionally, subvisible particles were analysed by BMI, and the monomer content, HMW species, and LMW species were determined via UP-SEC.

**Figure 4:**
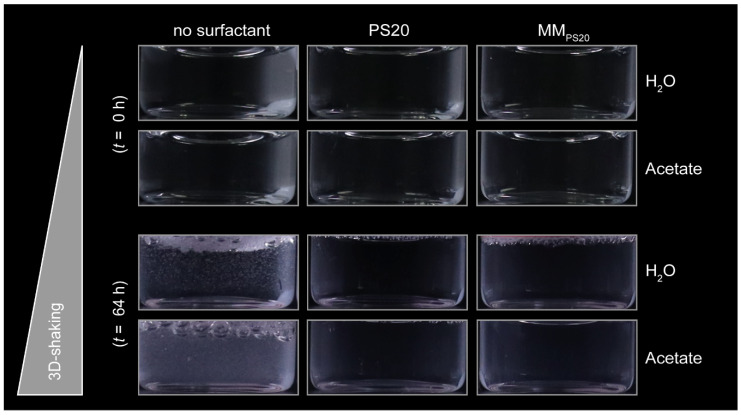
Visual inspection of the antibody shaking study with MMs. mAb1 (10 mg·mL^−1^) was formulated in water and 25 mM acetate buffer, pH 5.5, and was spiked without surfactant, with PS20 as well as with MMs composed of the phospholipid SPC3 (wf_SPC3_ of 0.3) and PS20 (MM_PS20_). All formulations were shaken for 64 h at RT. The surfactant concentration was 0.2 mg·mL^−1^. Visual inspection images were taken initially (*t* = 0 h) and after shaking (*t* = 64 h). The vials are 16 mm in width.

VI results before and after shaking of mAb1 in water and 25 mM acetate buffer, pH 5.5, are illustrated in Figure 4. Independent, whether formulated in water or an acetate buffer, no visual phenomena were observed initially (*t* = 0 h). After 64 h of 3D-shaking, formulations without surfactant are turbid, and small particles can be observed in water and a turbid solution for the acetate pH 5.5 formulation (Figure 4). However, the positive control containing 0.2 mg·mL^−1^ PS20 and the MMs containing PS and SPC3 with weight fractions of 0.3 (MM_PS20_) showed no visual phenomena, and no particles were detected under the tested conditions (Figure 4).

Transmission and turbidity measurements were performed to complement the VI results. Transmission (*n* = 2) and turbidity (*n* = 2) for 10 mg·mL^−1^ mAb1 in water and 25 mM acetate buffer, pH 5.5, before and after 40 h and 64 h shaking with and without surfactant are summarised in Table 1. In general, in the absence of surfactants, a decrease in transmission and an increase in turbidity are observed for shaken samples of mAb1 in acetate buffer and water. In contrast, a comparably slight decline in transmission and a slight increase in turbidity were detected for formulations containing PS20 or MMs. Without surfactant, the turbidity increased in water and acetate buffer after 64 h of shaking to 15 FNU and 104 FNU, respectively (Table 1). For samples formulated in water and the presence of PS20, no notable increase was detected under the tested conditions as the turbidity increased from 0.6 FNU to 1.5 FNU within 64 h of shaking, which falls within the expected measurement variability (Table 1). For the mixed micellar system composed of SPC3 (wf_SPC3_ of 0.3) and PS20 formulated in water, turbidity increased from 0.9 FNU to 8.5 FNU after 64 h of shaking (Table 1). The same trend could be observed for the acetate formulations. However, higher absolute values in turbidity were measured after 64 h of shaking, with 104 FNU, 6 FNU, and 18 FNU for formulations without surfactant, with PS20, and with MMs, respectively (Table 1). In the acetate buffer pH 5.5, all samples initially showed higher turbidity values of 5 FNU than the initial water turbidity values (0.6–0.9 FNU). These results align with the VI observations, showing samples with slightly higher turbidity (compare Table 1 and Figure 4).

**Figure 5:**
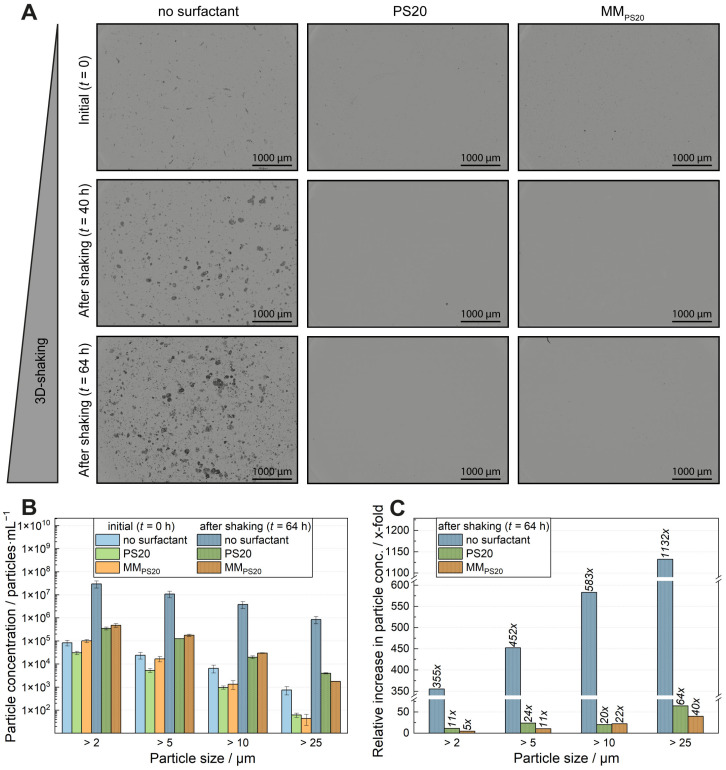
Results of particle analysis after shaking of an antibody in the absence and presence of surfactants (PS20 or MM_PS20_). mAb1 was formulated in a 25 mM acetate buffer, pH 5.5, and was investigated without surfactant (no surfactant), spiked with PS20 or MMs composed of the PL SPC3 (wf_SPC3_ of 0.3) and PS20 (MM_PS20_). All formulations were shaken for 40 h and 64 h at RT (*n* = 3, two independent MM productions and two independent particle measurements). The surfactant concentration was 0.2 mg·mL^−1^. (**A**) Brightfield images before (*t* = 0) and after shaking for 40 h and 64 h. (**B**) Particle concentrations (particles per mL) in logarithmic scale for particle sizes of >2 µm, >5 µm, >10 µm, and >25 µm before and after shaking (64 h) were determined via BMI. Columns without pattern show initial values, whereas columns with pattern show particle concentrations after shaking (64 h) for each particle size range. (**C**) Particle concentration increases relative to the initial for particle size ranges of >2 µm, >5 µm, >10 µm, and >25 µm.

To validate turbidity measurements, particle numbers and sizes were assessed before and after 40 h and 64 h of shaking using BMI. Formulations without surfactants, with PS20, and with MMs (wf_SPC3_ of 0.3) at 0.2 mg·mL^−1^ in acetate buffer pH 5.5 were analysed. Figure 5A shows background membrane images at different shaking times. Samples without surfactants exhibited a marked increase in visible particles over time, whereas those with PS20 or MMs did not exhibit a comparable increase under the tested conditions. A small number of particles was initially present in both the surfactant-free and MM-containing samples.

Figure 5 presents particle concentrations for size ranges >2 µm, >5 µm, >10 µm, and >25 µm before (*t* = 0) and after 64 h of shaking. Initially, PS20 and MM_PS20_ formulations showed low counts of particles > 25 µm (approx. 60 and approx. 40 particles∙mL^−1^, respectively), while the surfactant-free sample had approx. 750 particles∙mL^−1^. The highest total particle count (>2 µm) at *t* = 0 was observed in MM_PS20_ samples under the tested conditions (approx. 100,000 particles∙mL^−1^), followed by surfactant-free (approx. 84,000) and PS20 (approx. 31,000). These findings align with the brightfield images: large particles were visible without surfactants, and many small ones were in the MM_PS20_ samples.

After 64 h, particle concentrations increased in all formulations, with the largest rise in surfactant-free samples. For particles > 2 µm, over 30,000,000 particles∙mL^−1^ were detected without surfactant, compared to approx. 350,000 (PS20) and approx. 477,000 (MM_PS20_). This trend was generally observed across the tested size ranges. MM_PS20_ samples generally showed slightly higher particle counts than PS20, except for particles > 25 µm, where PS20 revealed a higher particle amount. However, MM_PS20_ samples exhibited higher baseline levels initially, as seen in Figure 5B.

To isolate the effect of shaking, relative increases were calculated (Figure 5C). Surfactant-free formulations showed the highest fold increases across all sizes: 355-fold (>2 µm), 452-fold (>5 µm), 583-fold (>10 µm), and 1132-fold (>25 µm). PS20 and MM_PS20_ also demonstrated their highest relative increases for particles > 25 µm (approx. 65-fold and approx. 40-fold, respectively). The lowest increase (>2 µm) was observed for MM_PS20_ (approx. 5-fold). PS20 formulations generally exhibited higher relative increases than MM_PS20_, except for particles > 10 µm.

In absolute terms, MM_PS20_ samples presented higher particle counts than PS20 for all sizes < 25 µm after 64 h (1.4–1.5× higher), while PS20 revealed 2.3× more particles > 25 µm (Figure 5B). The net increase in particle numbers (final minus initial) is shown in Supplement Figure S2. MM_PS20_ formulations showed greater increases for particles > 2 µm, >5 µm, and >10 µm, while PS20 exhibited around 4000 newly formed particles > 25 µm compared to approximately 1700 particles in MM_PS20_.

**Figure 6:**
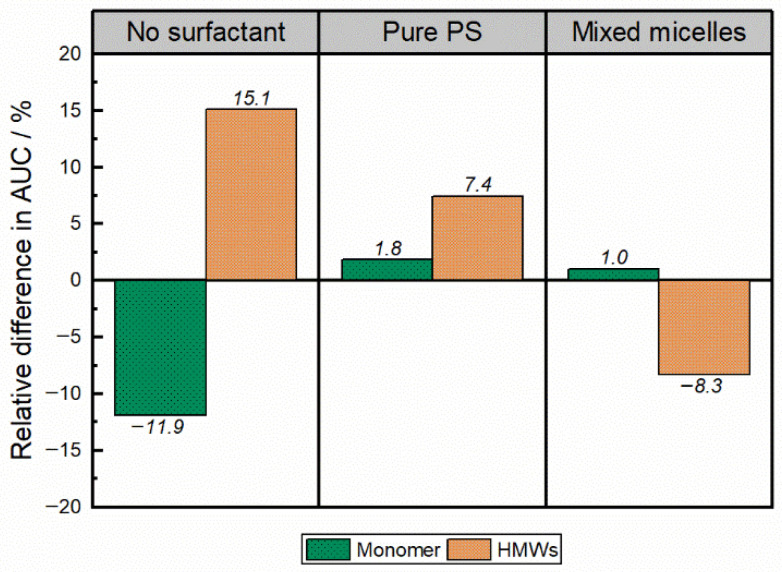
Chromatographic characterisation of an antibody before and after shaking. Size-exclusion chromatography measurements were performed before and after shaking (64 h) of mAb1 (10 mg·mL^−1^) formulated in 25 mM acetate buffer, pH 5.5, with or without the use of surfactants. A surfactant concentration of 0.2 mg·mL^−1^ was used, and the MMs were composed of the phospholipid SPC3 (wf_SPC3_ of 0.3) and PS20 (MM_PS20_). The relative differences in the monomer and HMWs AUCs before and after 64 h of shaking are shown.

UP-SEC was used to evaluate the protein stability by determining monomer, HMW, and LMW species content. Samples with 10 mg·mL^−1^ mAb1 (i) in the absence of surfactants and (ii) in the presence of 0.2 mg·mL^−1^ PS20 or (iii) MMs (PS20 and SPC3 wf_SPC3_ of 0.3) were investigated. Figure 6 illustrates the difference in monomer content and the amount of HMW species for acetate-buffered samples (pH 5.5) compared to the initial, indicating clear differences between samples in the presence or absence of PS20/MMs (surfactant). The relative difference in the monomer and HMW AUCs before and after 64 h of shaking is plotted in Figure 6. A strong monomer decrease of −11.9% after 64 h of shaking was detectable for samples without PS20/MMs (Figure 6, no surfactant). In contrast, in PS20 and MM_PS20_ formulations, no notable decrease in the monomer content was detected under the tested conditions (Figure 6). The HMW content determination showed comparable trends to those observed for the monomer content. In the absence of PS20/MMs (no surfactant), the HMW fraction increased by 15.1% after 64 h of shaking compared to the corresponding initial value (Figure 6). However, in the presence of PS20, the increase in the HMWs was considerably reduced (7.4%). In the presence of MM_PS20,_ a slight reduction was detected in the initial HMWs AUC after shaking (−8.3%).

## 4. Discussion

### 4.1. Mixed Micelles Produced by an Optimised Direct Dispersion Method (5-2-2 Method)

We adapted and optimised the direct dispersion method of Rupp et al. through a larger screening assay with different temperature and time profiles. The best formulations in the shortest time were achieved by adding a second heating step after the equilibration phase (exceeding the phase transition temperature of the used PL) [50,61]. The procedure is referred to as the 5-2-2 method. The phase transition temperature of SPC3 has been determined upfront by DSC measurements (Supplement Figure S3). Following, 55 °C was chosen for the increased temperature steps to exceed the phase transition temperature of the used PL SPC3. A temperature above the T_m_ of the PL is important to promote hydration and effective solubilisation of the lipid for successful MM production [71]. Thereby, MMs of SPC3 and PS20 were successfully produced without the use of organic solvents and within only 9 h for different weight fractions of PL (wf_SPC3_ of 0.3 to 0.6) and different total MM concentrations (5 mg·mL^−1^ to 50 mg·mL^−1^). Their formation was monitored by transmission and particle size measurements (Figure 1). MM production typically results in isotopically clear solutions and monodisperse size distributions, showing that the corresponding lipid is effectively solubilised within the micelle. The role of the second heating step was demonstrated by transmission and DLS measurements, as otherwise turbid solutions or multimodal particle size distributions were obtained (Figure S1 and Figure 1). Turbidity can be detected by the human eye starting at approximately 5 NTU [61,68,72]. To correlate NTU values with transmission values, the transmission of formazin standards was measured. A transmission value of 98.7% was calculated for a turbidity of 5 NTU. Hence, transmission values below 99% were not considered as clear solutions.

Using the method of Rupp et al. [50,61], only transmission values < 91% were detected (for 50 mg·mL^−1^ at a wf_SPC3_ of 0.4), whereas transmission values of >99% were determined after heating up to 55 °C a second time (compare Supplement Figure S1). High transmission values indicated reduced non-solubilised PLs due to lower light scattering, with the method’s feasibility limits reached at high surfactant concentrations combined with high SPC3 fractions (50 mg·mL^−1^ surfactant and wf_SPC3_ of 0.5 and 0.6). MM_PS20_ solutions got turbid due to insoluble PL proportions. These limitations were expected since the total surfactant concentration and ratio are important for promoting MM formation under the tested conditions [61,73]. At a total surfactant concentration of 50 mg·mL^−1^ with wf_SPC3_ of 0.5 and 0.6, the PS20 concentration is not sufficient for complete solubilisation of the phospholipid, resulting in increased turbidity. In contrast, for total MM concentrations of up to 20 mg·mL^−1^ with a wf_SPC3_ up to 0.5, as well as total concentrations of 50 mg·mL^−1^ with wf_SPC3_ up to 0.4, DLS analysis revealed monomodal size distributions with low PDI values (Figure 1). The PDI, ranging from 0 to 1, indicates the width and diversity of a particle size distribution. In our results, the PDI for the whole particle size distribution is presented, showing one overall average polydispersity that was calculated from a two-parameter fit to the correlation data, with a value of 1 reflecting a broad or multimodal distribution [74,75]. Consistent with high transmission values, low PDIs ranging between 0.07 and 0.2 with diameters of approximately 15–20 nm ± 3–5 nm were obtained for all tested conditions, except for 50 mg·mL^−1^ with wf_SPC3_ 0.5, as well as for all total surfactant concentrations in combination with wf_SPC3_ 0.6 (see Figure 1). PDIs < 0.1 illustrate monodisperse particle size distributions [74]. However, values up to 0.2 were considered as monodisperse as well [75,76]. It must be mentioned that this may as well be a bimodal distribution with one dominating species. Therefore, it should be remembered that DLS measures the fluctuation of light. In addition to diffusion-induced changes within the light intensity, other processes, such as fast micelle-forming and micelle-disintegration processes, could also contribute. Small differences were observed regarding the limit of successful MM production when comparing transmission values and DLS data. For total surfactant concentrations of 5 mg·mL^−1^ and 10 mg·mL^−1^ with wf_SPC3_ 0.6, high PDI values were detected (>0.4), indicating polydisperse systems or anisotropic structures (such as worm-like micelles) [77]. However, transmission values of 99% were determined after completing the 5-2-2 method. A similar outcome between transmission data and DLS analysis for all samples was not expected, as the light scattering intensity depends on the particle size [78]. Large particles of non-solubilised lipids drastically affect the scattering intensity within DLS measurements. For example, the light scattering intensities of 100 nm particles are 10^6^ times higher compared to 10 nm particles [78]. On the one hand, this can lead to an underestimation of the proportion of small particles. On the other hand, even the most negligible amounts of larger particles can be detected using intensity-weighted DLS data, allowing monomodal distributions to be evaluated reliably. In contrast, volume-weighted particle size distributions can be calculated from intensity-weighted distributions using Mie theory [79]. The additional evaluation of volume-weighted particle size distributions can prevent the extreme overrating of larger particles. Therefore, a comparison between volume-weighted particle size distributions and intensity-weighted particle size distributions is presented in Supplement Figure S4. The additional peaks visible in the intensity-weighted data for mixed micellar systems with total concentrations of 5 mg·mL^−1^ and 10 mg·mL^−1^ with wf_SPC3_ 0.6 appear noticeably smaller or almost non-existent in the volume-weighted data. In contrast to intensity-weighted particle size distributions, 10-fold larger particles show 1000-fold higher values in volume-weighted size distributions. This gives the impression that differences observed between high transmission values and a multimodal size distribution within intensity-weighted DLS data appear less pronounced when volume-weighted size distributions are considered. Nevertheless, the PDI of >0.4 remains, indicating a polydisperse particle size distribution despite transmission values of >99%. Therefore, merging both results might be suitable for reasonable evaluation after preparing MMs with the 5-2-2 method.

Combining these results from size and turbidity measurements, the feasibility limit of the 5-2-2 method of PS20/SPC3 systems can be approximated at a maximum of 20 mg·mL^−1^ total surfactant concentration with wf_SPC3_ 0.5 and at a maximum of 50 mg·mL^−1^ with wf_SPC3_ 0.4 under the tested conditions. After exceeding these concentrations and weight fractions, PS appears insufficient to completely solubilise the PL under these conditions, which may result in multimodal size distributions and higher turbidity, as discussed above.

### 4.2. Evidence of the Formation of Mixed Micelles Instead of Liposomal or Vesicular Structures

A dilution series of the MMs was examined by DLS (Figure 3A,B) and a Nile Red fluorescence method (Figure 3C) to determine the presence of MM in diluted samples. With a hydrodynamic radius of approximately 6–12 nm, the observed structures are MMs rather than vesicular structures [61]. Vesicles with radii of 6–12 nm are unlikely due to their high curvature [80]. It is noticeable that the radii remained around 8 nm for MM_PS20_ for a broad range of concentrations and weight fractions of PS20/SPC3, which was not observed for MM systems of bile salt and lecithin [81]. This can be taken as an indicator, suggesting that the 5-2-2 method supports the formation of a stable mixed-micellar system over a broad concentration range.

The experiments showed that MM_PS20_ appears stable over a wide concentration range, especially in dilute solution, and continues to exist as MMs. The observations from the DLS experiments (Figure 3A,B) were confirmed by the experiments with Nile Red, carried out as described by Bollenbach et al. [67]. A hydrophobic compartment could also be detected over the concentration range in which hydrodynamic radii typical of MMs were measured (Figure 3C). The combination of the two methods supports the assumption that MMs are present in the solution even after dilution to concentrations down to 0.02 mg·mL^−1^.

### 4.3. Prevention of Shaking Stress-Induced Protein Aggregation

Particle formation is a common issue within biopharmaceutical formulations since they can be generated through exposure of proteins to interfaces (air/water) or other hydrophobic surfaces. These physical stresses can occur during manufacturing or transportation [82,83,84]. Therefore, surfactants such as PSs are usually used to help mitigate protein particle formation [85]. The potential of MMs composed of the PL SPC3 and PS20 to support protein stability of BSA and mAb1 under stress conditions was evaluated by performing shaking studies.

Shaking studies are extreme stress conditions, as the proteins are extensively exposed to alternating interfaces, such as air/liquid or glass/liquid, resulting in protein unfolding and particle formation [69,70,86,87]. It was shown that the filling volume with or without a headspace leads to different results after shaking a PS20-free protein solution [70]. To enhance the interaction with hydrophobic interfaces, vials were filled in half with sample solutions to generate a large headspace and increase the interaction with hydrophobic interfaces for mAb1 solutions [69,70]. As albumin-containing solutions were used as initial trials, these had a filling volume of 2.5 mL in 2R vials.

### 4.4. Shaking Studies with BSA

BSA was used as a model protein to evaluate the protective stabilising effect of MMs (20 mg·mL^−1^) composed of the PL SPC3 and PS20 in acetate buffer at pH 5.5. Usually, the BSA monomer has a hydrodynamic diameter of 10–13 nm [88]. Before shaking, both tested BSA-MM formulations showed a small percentage of larger particles (about 600–700 nm). After shaking without surfactant, a multimodal particle size distribution appeared with two additional peaks (25–700 nm), indicating the formation of larger particles (Figure 2). In contrast, the addition of MM_PS20_ led to comparable particle size distributions as observed before shaking, supporting the indication of a protective effect. For samples containing MM with a wf_SPC3_ of 0.3, only minor changes were detected in particle size distribution under the tested conditions. A similar behaviour was observed for samples containing an MM_PS20_ weight fraction of 0.4. As described above, albumin has an approximate hydrodynamic diameter of 10–13 nm [88], while MMs of PS20 show sizes of 15–20 nm, masking the albumin peak. It is worth mentioning that intensity-weighted distributions were used and analysed to detect small changes in particle size, as larger particles scatter drastically higher amounts of light. In general, the albumin shaking study in acetate buffer was used as the first hint in evaluating the capabilities of MMs in protein stabilisation. It must be mentioned that within these initial trials, high MM concentrations were used (20 mg∙mL^−1^). This would not be the case within final biological formulations. Nevertheless, these results were used as preliminary experiments, suggesting that MMs may help mitigate particle formation in antibodies. Subsequently, a shaking study with a monoclonal antibody was performed in a lower, more formulation-relevant concentration to further investigate the potential protective function of MMs composed of PL and PS20.

### 4.5. Shaking Studies with Monoclonal Antibodies

It is important to evaluate the protective effects of MM for therapeutic antibodies during shaking to establish the practical relevance of MMs as suitable additives. Consequently, a shaking study with mAb1 at 10 mg·mL^−1^ was performed in the presence of PS20, MM_PS20,_ and in the absence of surfactant in water and acetate buffer at pH 5.5. The surfactants were used in a concentration of 0.2 mg·mL^−1,^ and the MMs contained lipid weight fractions of 0.3. VI, turbidity, particle counts, and sizes via BMI, and UP-SEC were performed before and after shaking to evaluate the protective effect of the mixed micellar solution.

VI revealed that no visible particles were detected in the presence of either PS20 or MM_PS20_ in water and acetate buffer at pH 5.5. On the contrary, visible particles were observed for both formulation conditions in the absence of surfactant. These results suggested a protective effect of the mixed micellar system. Turbidity measurements, enabling detection of protein particles < 50 µm, supported the VI results for both formulations. Without surfactant, turbidity increased after 64 h of shaking in acetate buffer (104 FNU) and water (15 FNU), consistent with VI results (Table 1). Slightly higher turbidity values were observed in the MM_PS20_ formulation compared to the PS20 formulations after shaking, suggesting slightly better protection of the PS20 formulations.

Particle analysis was performed via BMI to evaluate emerging subvisible particles. Brightfield images revealed clear differences with and without surfactant (Figure 5A), confirmed by higher particle concentrations across all subvisible particle sizes in the absence of surfactant. In contrast, formulations containing PS20 or MM_PS20_ show a slight increase in particles for all measured particle sizes. A more pronounced increase in particles was detected for formulations containing MM_PS20_ (Figure 5B). However, higher particle concentrations for formulations containing MM_PS20_ were found initially. Therefore, the relative increase in particles was calculated to evaluate the emerging particles upon shaking stress (Figure 5C). Higher particle concentrations for MM_PS20_ formulations were also observable in the brightfield images. The MMs themselves most likely cause these small particles. The protective effect of the MM systems for shaking should be evaluated without pre-existing particles before shaking, so absolute particle concentrations could be misleading. Comparing the relative increase in particles over all measured sizes, a higher increase in shaking-induced particles was determined for PS20 formulations compared to MM_PS20_ samples. The relative increase for mixed micellar formulations was around half of the relative increase in the PS20-containing formulations for particle sizes of >2 µm and >5 µm and around two-thirds for particle sizes > 25 µm (Figure 5C), indicating a protective effect of MM_PS20_. Only for particles > 10 µm, mixed micellar formulations showed a little higher relative increase than PS20-containing formulations (Figure 5C). The higher absolute particle concentrations after shaking formulations containing MM_PS20_ for 64 h occur presumably because the initially measured particle count was higher than that of the initially measured particle counts for PS20-containing formulations. The absolute and relative particle increase would have been higher than in PS20-containing formulations if the mixed micellar system could not protect the antibody from particle formation. Since the opposite was measured, the mixed micellar system had a beneficial effect.

Finally, the mAb shaking study was evaluated by UP-SEC to get information on HMWs induced by shaking. A monomer loss of approximately 15% was detected after 64 h of shaking in the absence of surfactant (Figure 6). With PS20 or MM_PS20_ being present, no notable monomer loss was detected, revealing their protective effect. A similar protective trend was observed for the HMW species, which did not exhibit a notable increase (Figure 6). A small decrease in the HMW fraction of the formulation containing MM_PS20_ after 64 h of shaking was found, resulting in a HMW fraction decrease of −8.3%. Nevertheless, both formulations can protect the investigated mAb against the formation of HMWs.

Considering the performed analysis portfolio before and after shaking, our data suggest that the mixed micellar system composed of SPC3 and PS20 helps mitigate HMWs formation of mAbs in a way comparable to PS20. Our results demonstrated that non-toxic, endogenous, and surface-active PLs can generate MMs with PS20. Therefore, it was possible to reduce the effective concentration of PS20 in the formulation while maintaining protein-stabilising properties under the tested conditions.

In our investigations, a monoclonal antibody and BSA were tested as model proteins. For both tested proteins, a protective effect of MM_PS20_ was observed. Therefore, mixed micellar systems composed of SPC3 and PS20 could potentially serve as a surfactant alternative to help mitigate protein instability.

## 5. Conclusions

In recent years, protecting proteins against high molecular weight species and particle formation has become an increasingly important task in research and development departments of the pharmaceutical industry. While alternative surfactants may be used, another innovative strategy would be to minimise PS in biopharmaceutic formulations by supplementing it with other surfactants capable of forming mixed micellar systems. MMs nowadays are mainly used to solubilise poorly water-soluble small-molecule drugs instead of stabilising antibodies or other proteins.

Here, we describe an optimised direct dispersion method for the production of MMs composed of PLs and PS20 by introducing a second heating step above the lipid phase transition temperature. These MM systems can be manufactured over a broad range of total concentrations and different compositions of PS20 and the PL SPC3, which typically exhibit monodisperse particle sizes of approximately 15 nm and high transmission values > 99%. No toxicologically harmful solvents are needed. The limit of feasibility of the 5-2-2 method was estimated under the tested conditions to be at a maximum of 20 mg·mL^−1^ total concentration for PL contents not higher than 50% and a maximum of 50 mg·mL^−1^ for PL contents not higher than 40%. Exceeding these limits appears to limit the effective solubilisation of the remaining PL. The tested concentration range of 50 mg·mL^−1^ is significantly higher than the commonly used concentration of surfactants (0.01–2 mg·mL^−1^) required for protein protection in biopharmaceutical formulations [89]. Hence, these MM solutions produced with the 5-2-2 method may also be suitable for solubilising poorly soluble proteins and thus are versatile in use.

Secondly, for the first time, the protective effects of MMs composed of PS20 and PLs against stress-induced protein-aggregation were shown. Shaking studies were performed with BSA and mAb1 in the presence of the MM system, with PS20, and without surfactant. A comparable performance in protecting a monoclonal antibody against shaking stress-induced aggregation was observed between PS20 and the MM system with a 30% reduced PS content, suggesting a potential protective effect by MM on a monoclonal antibody for the first time. These MM solutions and the 5-2-2 method are a novel alternative to the currently commonly used surfactants for helping mitigate protein particle formation. Future studies should further evaluate the mixed micellar system with protein therapeutics and its use in biopharmaceutical formulations.

## Figures and Tables

**Table 1:** Transmission and turbidity data after shaking mAb1 (10 mg·mL^−1^) in water and 25 mM acetate buffer, pH 5.5, without surfactant, with PS20, and with MMs composed of SPC3 (wf_SPC3_ of 0.3) and PS20. The surfactant concentration was 0.2 mg·mL^−1^. Data (transmission and turbidity) were obtained for two biological replicates (*n* = 2), each with two technical replicates.

		Transmission/%			Turbidity/FNU		
		Initial	40 h	64 h	Initial	40 h	64 h
mAb1 in water	without surfactant	101.4	67.3	76.5	0.6 ± 0.02	20 ± 1.27	15 ± 0.71
	with PS20	97.3	93.2	88.8	0.6 ± 0.04	1 ± 0.38	1.5 ± 0.10
	with MM_PS20_	103.9	93.9	90.0	0.9 ± 0.01	8 ± 0.42	8.5 ± 1.07
mAb1 in acetate buffer pH 5.5	without surfactant	94.3	51.3	48.7	5 ± 0.05	83 ± 16.3	104 ± 5.08
	with PS20	99.8	91.4	83.6	5 ± 0.03	6 ± 0.44	6 ± 0.48
	with MM_PS20_	103.9	91.7	85.6	5 ± 0.02	16 ± 1.09	18 ± 2.12

## Data Availability

The original contributions presented in this study are included in the article/Supplementary Material. Further inquiries can be directed to the corresponding author.

## Supplementary Materials

The following supporting information can be downloaded at: https://www.mdpi.com/article/10.3390/pharmaceutics18030321/s1, Figure S1: Transmission analysis of the mixed micelle production with an additional second heating step to 55 °C; Figure S2: Particle analysis via Horizon after shaking of an antibody in the absence and presence of surfactants (PS20 or MM_PS20_); Figure S3: Transition temperature of the used SPC3 lipid determined with differential scanning calorimetry; Figure S4: Intensity and volume weighted particle size distributions of mixed micelles in acetate buffer.

## Abbreviations

BMI	Background membrane imaging
BSA	Bovine serum albumin
CMC	Critical micelle concentration
DLS	Dynamic light scattering
DSC	Differential scanning calorimetry
FNU	Formazin nephelometric unit
HMW	High molecular weight
LMW	Low molecular weight
mAb	Monoclonal antibody
MM	Mixed micelle
MM_PS20_	MMs containing polysorbate and SPC3 with weight fractions of 0.3
NTU	Nephelometric turbidity unit
P188	Poloxamer 188
PDI	Polydispersity index
PL	Phospholipid
PS	Polysorbate
PS20	Polysorbate 20
PS80	Polysorbate 80
SEC	Size-exclusion chromatography
SPC3	Soybean phosphatidylcholine with an iodine value of 3
*T*_m_	Gel-to-liquid phase transition temperature
UP-SEC	Ultra-performance size-exclusion chromatography
VI	Visual inspection
wf	Weight fraction
