# Supplementary material for: Phosphatidylcholine-Polysorbate 20-Based Mixed Micelles: A New Option to Prevent Protein Aggregation?
Source: Pharmaceutics. 2026 Mar 2;18(3):321. doi: 10.3390/pharmaceutics18030321 (PMC13029507; doi:10.3390/pharmaceutics18030321)
Supplement: Supplementary file 1 [file pharmaceutics-18-00321-s001.zip › pharmaceutics-4129661-supplementary.pdf]

pharmaceutics

MDPI

Supplementary Materials:

Phosphatidylcholine-Polysorbate 20—Based Mixed Micelles: A New Option to Prevent Protein Aggregation?

Johanna Weber, Tim Diederichs, Lukas Bollenbach, Patrick Garidel and Karsten Mäder

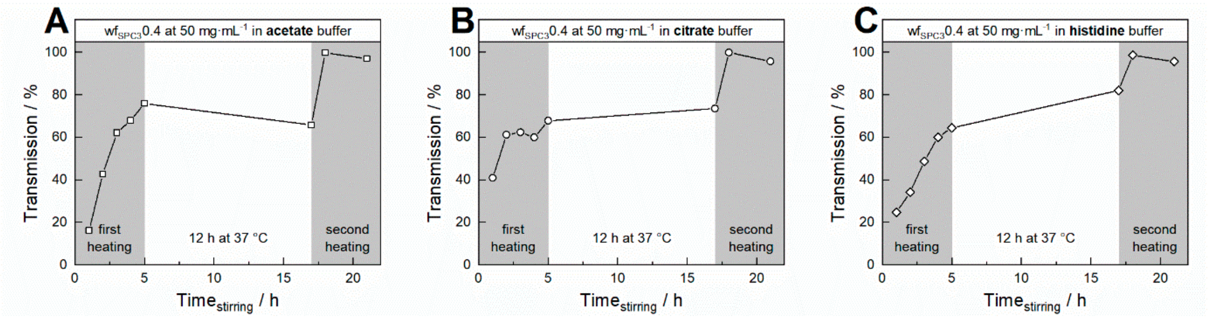

Supplementary Figure S1: Transmission analysis of the mixed micelle production with an additional second heating step to 55 °C. Mixed micelles composed of PS20 and phospholipid (SPC3) for a weight fraction of the lipid of 0.4 and a total concentration of 50 mg·mL $^{-1}$ were prepared by the described method from Rupp et al. (2010) and an additional heating step to 55 °C was performed. The timeframes at 55 °C are highlighted in light grey. Mixed micelles were prepared in three different buffer systems at pH 5.5. **(A)** Transmission values of mixed micelle solutions determined in acetate buffer, **(B)** Transmission values of mixed micelle solutions determined in citrate buffer **(C)** Transmission values of mixed micelle solutions determined in histidine buffer.

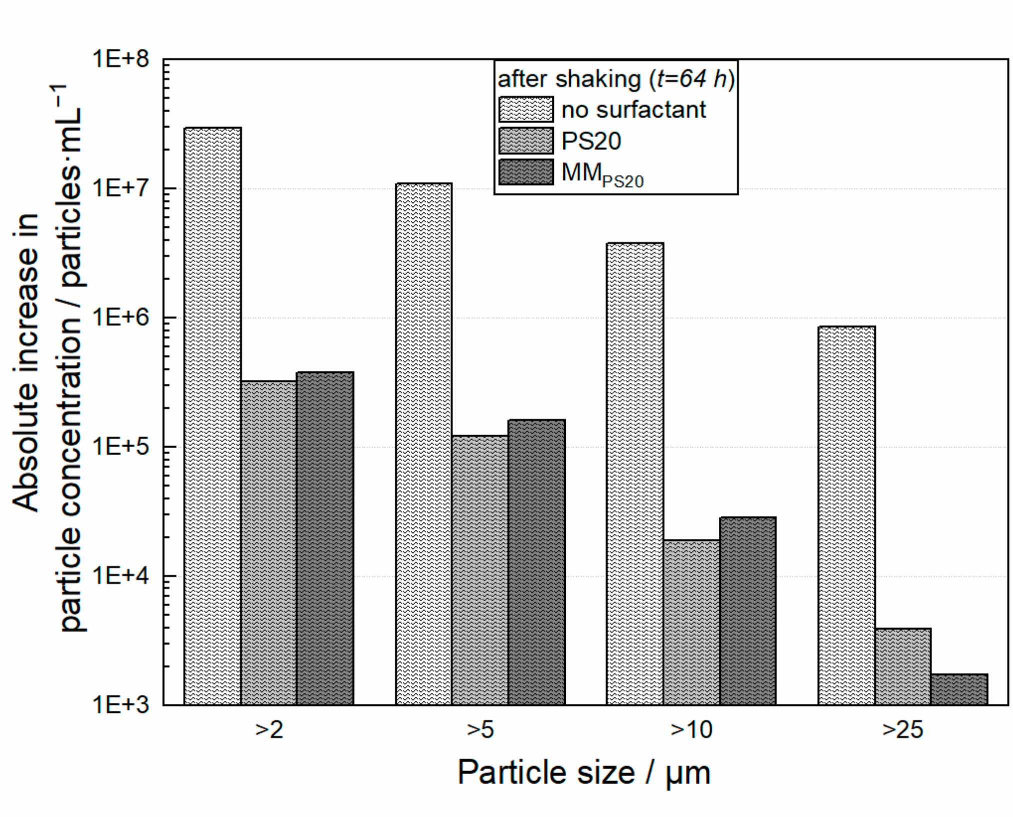

Supplementary Figure S2: Particle analysis *via* Horizon after shaking of an antibody in the absence and presence of surfactants (PS20 or MM_{PS20}). The mAb was formulated in a 25 mM acetate buffer, pH 5.5, and was spiked with mixed micelles composed of the phospholipid SPC3 (wf_{SPC3} of 0.3) and PS20 (MM_{PS20}), with PS20, as well as without surfactant, and was shaken for 40 and 64 h at RT. The surfactant concentration was 0.2 mg·mL^{-1}. The initially occurring absolute particle counts were subtracted from the absolute particle counts after agitation.

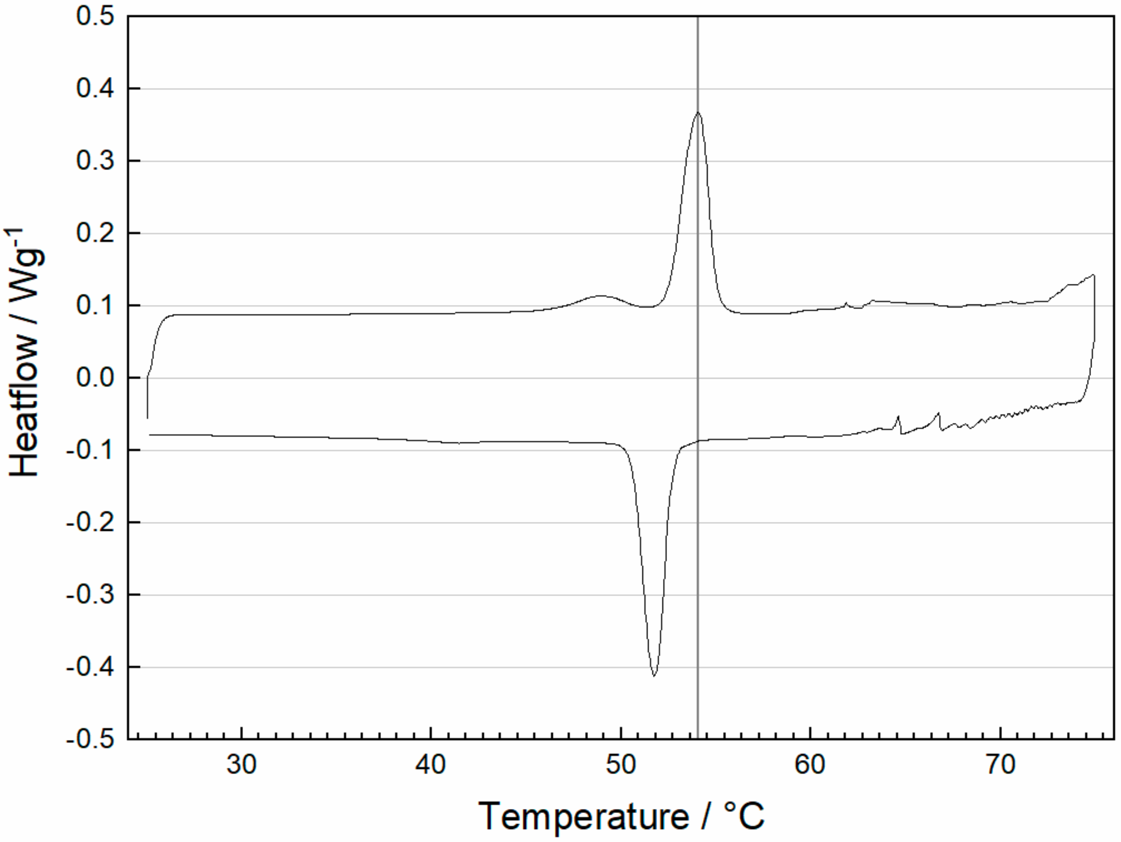

Supplementary Figure S3: Transition temperature of the used SPC3 lipid determined with differential scanning calorimetry. Determination of the phase transition temperature of SPC3. SPC3 was incubated in ultrapure water at ambient temperature for 30 minutes for swelling. Thereafter, it was homogenized for 2 minutes, at 14,000 rpm. The ratio of ultrapure water to SPC3 was set to 3:1. A closed aluminum pan was heated from 25 – 75 °C using a heating rate of 0.5 K·min^{-1} to characterise the thermal behavior of the SPC. The maximum of the second heating curve was identified as the phase transition temperature.

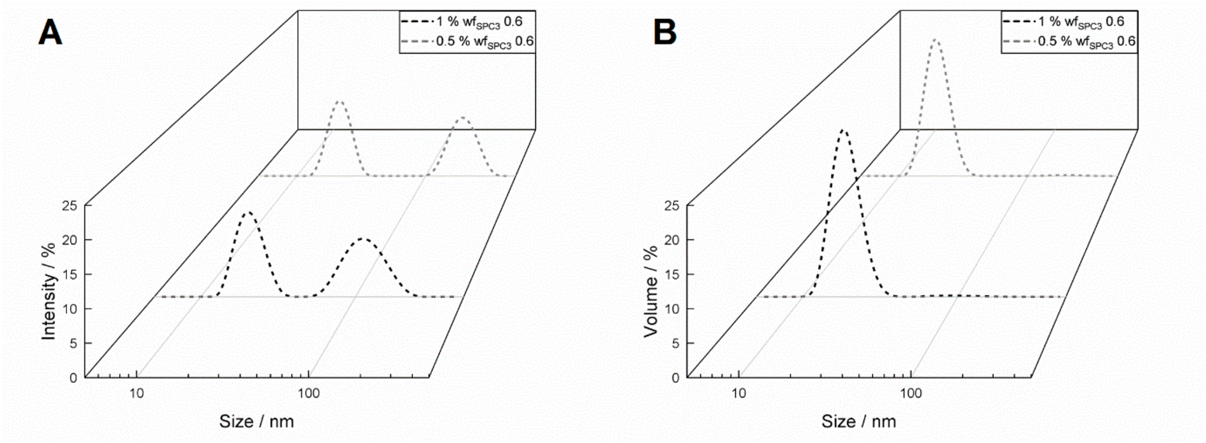

Supplementary Figure S4: Intensity and volume weighted particle size distributions of mixed micelles in acetate buffer. Acetate buffer and mixed micelles wf$_{SPC3}$ 0.6 in 0.5 % and 1 % total concentration were spiked together and were measured using DLS. Particle size distributions were analysed after finishing the 5-2-2 method. Intensity-weighted particle size distributions (**A**) and volume weighted particle size distributions are shown (**B**).
